# Effect of Statin Use on Mortality in Individuals With Cardiovascular–Kidney–Metabolic Syndrome: A Retrospective Propensity Score‐Matched Cohort Study

**DOI:** 10.1155/cdr/7480081

**Published:** 2026-03-31

**Authors:** Cheng Yuan, Yuguo Ge, Wenqi Ge, Huaxue Wang, Ximing Deng, Jinmeng Chen, Kaixuan Niu, Jinhui Xiong, Yang Xu, Kun Lu

**Affiliations:** ^1^ Department of Critical Care Medicine, The First Affiliated Hospital of Bengbu Medical University, Bengbu, China, bbmc.edu.cn; ^2^ Department of Critical Care Medicine, Tongling Hospital of Bengbu Medical University, Tongling, China; ^3^ Obstetrics and Gynecology, The First Affiliated Hospital of Bengbu Medical University, Bengbu, China, bbmc.edu.cn

**Keywords:** all-cause mortality, cardiovascular mortality, cardiovascular–kidney–metabolic syndrome, propensity score matching, statin

## Abstract

**Background:**

The present study is aimed at investigating the effects of statins on all‐cause and cardiovascular mortalities in individuals with cardiovascular–kidney–metabolic syndrome (CKMS).

**Methods:**

Using data from the National Health and Nutrition Examination Survey (NHANES) 1999–2018, we identified individuals with CKMS and categorized them into statin‐treated and untreated groups based on statin exposure. A propensity score matching (PSM) model was applied to balance baseline characteristics between the two groups. All‐cause and cardiovascular mortalities were then assessed.

**Results:**

A total of 19087 individuals with CKMS were enrolled, yielding 4762 matched individuals. Compared with the statin‐untreated group, statin therapy was associated with significant mortality benefits in CKMS individuals, demonstrating a 27% reduction in all‐cause mortality (HR 0.73, 95% CI 0.65–0.82) and a 26% decrease in cardiovascular mortality (HR 0.74, 95% CI 0.61–0.90). Stratified analyses by CKMS stage showed no significant interaction between disease stage and statin use for either all‐cause (*p* for interaction = 0.81) or cardiovascular mortality (*p* for interaction = 0.13), although statistically significant inverse associations were primarily observed in individuals with advanced CKMS stages. Analyses by statin type indicated that atorvastatin and simvastatin were associated with reduced risks of both all‐cause and cardiovascular mortalities, whereas rosuvastatin was associated with a reduced risk of all‐cause mortality only. Exploratory subgroup analyses suggested that the associations were more evident among individuals with intermediate levels of total cholesterol (200–240 mg/dL) and LDL cholesterol (130–160 mg/dL).

**Conclusion:**

In this nationally representative cohort, statin use was associated with lower risks of all‐cause and cardiovascular mortalities among individuals with CKMS. These findings warrant confirmation in prospective studies with larger sample sizes to further clarify potential heterogeneity across statin type, disease stages, and different lipid profiles.

## 1. Introduction

Cardiovascular–kidney–metabolic syndrome (CKMS) is an emerging clinical construct that elucidates the intricate pathophysiological interplay among metabolic disorders, chronic kidney disease (CKD), and cardiovascular disease (CVD) (1). Epidemiological data show that it is slowly becoming a major global health threat. In the United States, over 25% of adults met diagnostic criteria for CKMS, with more than half progressing to Stage 2 or higher disease severity, while disease control rates showed no improvement from 2011 to 2020 (2, 3). Italian cohort studies during the COVID‐19 pandemic revealed marked increases in hypertension, dyslipidemia, and obesity prevalence, alongside a 1.7‐fold surge in prediabetes incidence, leaving nearly 50% of the population with ≥ 1 CKMS‐related complication (4). Despite these concerning epidemiological trends, evidence‐based frameworks for multisystem CKMS management remain in their nascent stages, with current therapeutic approaches largely relying on extrapolated evidence from isolated disease guidelines rather than clinical studies specifically designed for CKMS.

Statins, as cornerstone agents for lipid management, have demonstrated pleiotropic benefits beyond cholesterol reduction, including anti‐inflammatory and endothelial‐stabilizing effects (5–7). While randomized trials have established the efficacy of statins in primary and secondary CVD prevention (8–10), real‐world evidence evaluating their role in CKMS is limited. Moreover, the differential mortality benefits of specific statin types in this population are poorly understood. Propensity score matching (PSM) analyses offer a robust method to address confounding biases inherent in observational data (11), yet such approaches have rarely been applied to assess statin therapy in CKMS.

To address these knowledge gaps, we conducted a PSM retrospective cohort study using National Health and Nutrition Examination Survey (NHANES) 1999–2018 data to investigate the association of statin treatment with all‐cause and cardiovascular mortalities in individuals with CKMS and examine heterogeneity in treatment effects across different CKMS stages and statin types. Our findings might provide timely insights into optimizing statin use within populations with CKMS, informing individualized therapeutic strategies.

## 2. Methods

### 2.1. Data Resource

We utilized data from the NHANES, a nationally representative study that systematically evaluates health status and nutritional profiles through comprehensive physical examinations and laboratory tests across various age groups in the United States. The NHANES protocol was approved by the Research Ethics Review Board of the National Center for Health Statistics (NCHS). As all data were fully deidentified, this study was granted exemption from institutional review board approval and waived the requirement for informed consent.

### 2.2. Study Population

Adult participants with CKMS were included in this study, with diagnosis and staging rigorously adhering to the American Heart Association (AHA) Presidential Advisory criteria, as detailed in Table S1. Additionally, CKMS was divided into 0–4 stages. Specifically, Stage 0 indicated no CKMS risk factors; Stage 1 comprised individuals with obesity‐related metabolic disturbances (including impaired glucose tolerance or prediabetes); Stage 2 was defined by the presence of ≥ 2 metabolic risk factors or moderate‐to‐severe CKD (Stages 3–4); Stage 3 represented subclinical CVD in the setting of metabolic abnormalities; and Stage 4 referred to established clinical CVD (encompassing coronary heart disease, heart failure, or stroke). The detailed staging criteria are provided in Table S2. In the staging of CKMS, Stages 0–2 are classified as nonadvanced, while Stages 3–4 are considered advanced (12).

### 2.3. Assessment of the Use of Statins and Outcome Events

Statin exposure was ascertained through participants’ self‐reports. Additionally, trained interviewers checked participants’ medication bottles to ensure accurate documentation of medication use. The type of statin contains atorvastatin, simvastatin, fluvastatin, lovastatin, pravastatin, rosuvastatin, and pitavastatin. Due to database limitations, we did not analyze detailed information on the dosage, duration of treatment, and medication adherence of statins.

The primary outcomes of this study were all‐cause mortality and cardiovascular mortality. Mortality data were derived from the publicly available linked mortality files of the NHANES, which were deterministically matched with records from the National Death Index. Causes of death were strictly classified according to the International Classification of Diseases, 10th Revision (ICD‐10) codes. All‐cause mortality encompassed all fatal cases, while cardiovascular mortality was explicitly defined as deaths attributable to CVD (I00–I09, I11, I13, and I20–I51) and cerebrovascular diseases (I60–I69). The follow‐up duration was calculated from the baseline survey date until the occurrence of death or the study endpoint. All raw data were obtained from the official repository of the Centers for Disease Control and Prevention (CDC) (https://wwwn.cdc.gov/nchs/nhanes/), with data collection and processing methodologies referenced from previously published protocols.

### 2.4. Covariates

We extracted data from the NHANES, including demographic characteristics, clinical measurements, laboratory biomarkers, lifestyle behaviors, and chronic disease status. Specifically, baseline demographic profiles encompassed age, gender, race/ethnicity, educational levels, and poverty‐to‐income ratio; anthropometric evaluations included body mass index (BMI), waist circumference (WC), and triplicate averaged systolic and diastolic blood pressure (SBP/DBP) measurements; laboratory biomarkers covered hepatic function (alanine aminotransferase [ALT] and aspartate aminotransferase [AST]), lipid metabolism (triglycerides [TGs], total cholesterol [TC], high‐density lipoprotein cholesterol [HDL‐C], and low‐density lipoprotein cholesterol [LDL‐C]), glucose metabolism (fasting plasma glucose [FPG] and hemoglobin A1c [HbA1c]), and renal function (estimated glomerular filtration rate [eGFR]); behavioral assessments documented smoking status and alcohol consumption, while chronic disease incorporated diagnosed hypertension, diabetes, and corresponding pharmacotherapy regimens. All variables were collected in strict accordance with standardized NHANES protocols. Missing data distributions are shown in Figure S1, with imputation via multiple imputation.

### 2.5. Statistical Analysis

The study cohort was dichotomized into statin‐treated and statin‐untreated groups. To address potential confounding, we performed PSM. Propensity scores were derived via logistic regression, estimating each patient’s probability of receiving statins. Matching used a 1:1 nearest neighbor algorithm (caliper width = 0.2 without replacement). Balance was validated using standardized mean differences (SMDs < 0.10).

Continuous variables were reported as mean ± standard deviation (SD) or median (interquartile range [IQR]), compared via *t*‐tests/Mann–Whitney *U* tests. Categorical variables were presented as counts (percentages) and analyzed by *χ* ^2^/Fisher’s exact tests. All‐cause and cardiovascular mortalities were assessed using multivariable Cox regression (reporting adjusted hazard ratios [HRs] with 95% CIs). The Kaplan–Meier method was used to assess the cumulative incidence of all‐cause mortality and cardiovascular mortality, with intergroup differences evaluated using log‐rank tests. Variables with a *p* < 0.05 in the univariate analysis of the matched cohort dataset were entered into the multivariable analysis model for adjustment, including DBP, diabetes, diabetes medications, HbA1c, TC, and LDL‐C (Table S3). Variance inflation factors (VIFs) for all adjusted variables were less than 5 (Figure S2), ruling out multicollinearity.

Subgroup analyses were conducted in the matched cohort based on prespecified stratification criteria, including age (< 65 years and ≥ 65 years), sex (female and male), TC levels (< 200, 200–240, and > 240 mg/dL), and LDL‐C levels (< 70, 70–100, 100–130, 130–160, and > 160 mg/dL). In sensitivity analyses, we excluded participants who died within 2 years of follow‐up to minimize survival bias from terminal illness or acute‐phase events for the primary findings. Additionally, to assess the impact of unmeasured confounders on the results, we calculated *E* values based on multivariable models to quantify the minimum strength of association of unmeasured confounders required to overturn the observed associations of statin use with the risk of death from all causes and cardiovascular death.

All statistical tests were two‐tailed, with *p* < 0.05 considered significant. Analyses were performed using R software (Version 4.4.2).

## 3. Results

### 3.1. Participant Selection

**Figure 1:**
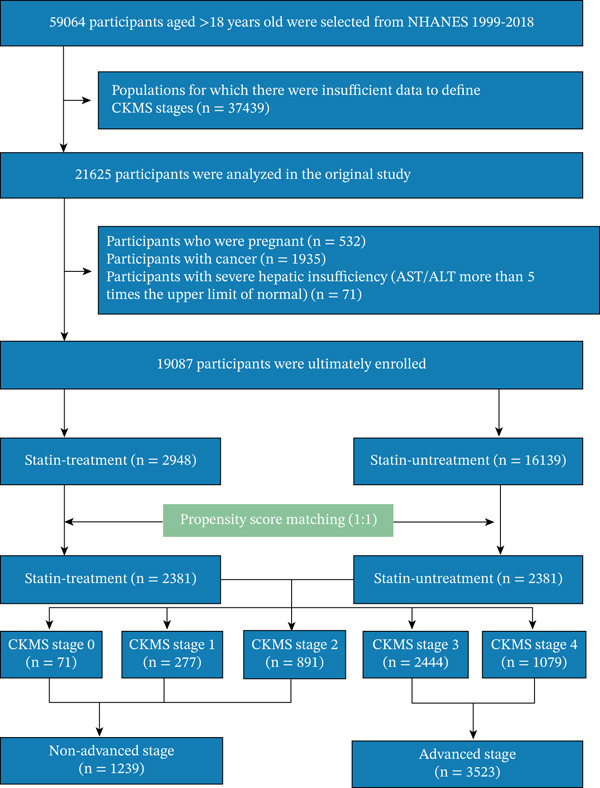
The flow chart of this study. NHANES, National Health and Nutrition Examination Survey; CKMS, cardiovascular–kidney–metabolic syndrome.

This study utilized data from the NHANES spanning 1999–2018. As shown in Figure 1, among 59,084 respondents aged 18 years or older, 21,625 participants who met the diagnostic criteria for CKMS (Stages 0–4) were identified. After excluding participants who did not meet the inclusion criteria, a total of 19,087 participants were enrolled. Of these, 2948 (15.45%) were receiving statin therapy at baseline. The average follow‐up time was 117.6 months. The PSM cohort comprised 4762 participants, with balanced groups of 2381 statin‐treated and 2381 statin‐untreated individuals, among whom 1239 (26.0%) had nonadvanced and 3523 (74.0%) had advanced CKMS stage.

### 3.2. Baseline Characteristics

Table 1 summarizes the baseline demographic and clinical characteristics of CKMS participants prior to and following PSM. In the overall cohort, statin‐treated participants were older, more likely to be male and White, reported higher proportions of never‐smokers and never‐alcohol consumers, and exhibited greater prevalences of hypertension and diabetes along with corresponding medication use. These participants also demonstrated higher FPG, HbA1c, and TG levels, while showing lower values for BMI, WC, ALT, TC, LDL‐C, HDL‐C, and eGFR (Table S3). PSM achieved covariate balance (absolute SMDs < 0.10; Table S3 and Figure S3), although some imbalance persisted for certain variables (SMD for TC = 0.143 and SMD for LDL − C = 0.164; Table 1). Figure S4 visually demonstrates the distributional balance before and after PSM.

**Table 1:** Baseline characteristics before and after propensity score matching.

Variables	Before propensity score matching			After propensity score matching		
	Statin‐untreated group	Statin‐treated group	SMD	Statin‐untreated group	Statin‐treated group	SMD
	*N* = 16,139	*N* = 2948		*N* = 2381	*N* = 2381	
Age (years old) (mean [SD])	45.56 (16.64)	64.41 (11.30)	1.325	63.35 (13.54)	62.96 (11.39)	0.032
Male, *n* (%)	8055 (49.9)	1553 (52.7)	0.055	1165 (48.9)	1217 (51.1)	0.044
Ethnicity, *n* (%)			0.223			0.032
White	6637 (41.1)	1467 (49.8)		1168 (49.1)	1178 (49.5)	
Black	3321 (20.6)	565 (19.2)		477 (20.0)	478 (20.1)	
Mexican	3205 (19.9)	374 (12.7)		344 (14.4)	319 (13.4)	
Other	2976 (18.4)	542 (18.4)		392 (16.5)	406 (17.1)	
Poverty‐to‐income ratio (mean [SD])	2.50 (1.62)	2.69 (1.61)	0.118	2.67 (1.61)	2.67 (1.63)	0.001
Education level, *n* (%)			0.081			0.025
Less than high school graduate	4391 (27.2)	838 (28.4)		690 (29.0)	669 (28.1)	
High school graduate or general equivalency diploma	3625 (22.5)	740 (25.1)		589 (24.7)	582 (24.4)	
Some college or above	8123 (50.3)	1370 (46.5)		1102 (46.3)	1130 (47.5)	
Blood pressure (mmHg)						
Systolic	122.36 (18.36)	129.96 (19.74)	0.399	131.15 (20.63)	130.02 (19.86)	0.056
Diastolic	70.91 (11.70)	68.08 (12.41)	0.235	69.71 (13.07)	68.85 (12.46)	0.068
Smoking status, *n* (%)			0.348			0.033
Never	8977 (55.6)	1398 (47.4)		1181 (49.6)	1142 (48.0)	
Former	3474 (21.5)	1082 (36.7)		819 (34.4)	845 (35.5)	
Now	3688 (22.9)	468 (15.9)		381 (16.0)	394 (16.5)	
Drinking status, *n* (%)			0.239			0.034
Never	2194 (13.6)	439 (14.9)		375 (15.7)	357 (15.0)	
Former	2503 (15.5)	719 (24.4)		580 (24.4)	559 (23.5)	
Now	11,442 (70.9)	1790 (60.7)		1426 (59.9)	1465 (61.5)	
BMI (kg/m^2^) (mean [SD])	28.59 (6.60)	30.08 (6.27)	0.232	30.04 (6.96)	29.95 (6.27)	0.013
WC (cm) (mean [SD])	97.29 (15.90)	104.33 (14.69)	0.460	103.55 (16.04)	103.60 (14.54)	0.003
Diabetes, *n* (%)	2032 (12.6)	1396 (47.4)	0.820	878 (36.9)	950 (39.9)	0.062
Hypertension, *n* (%)	5526 (34.2)	2179 (73.9)	0.868	1714 (72.0)	1712 (71.9)	0.002
Diabetes medications, *n* (%)	878 (5.4)	1044 (35.4)	0.801	568 (23.9)	659 (27.7)	0.087
Hypertension medications, *n* (%)	3125 (19.4)	2286 (77.5)	1.432	1684 (70.7)	1726 (72.5)	0.039
FPG (mg/dL) (mean [SD])	104.96 (33.04)	125.23 (45.60)	0.509	120.86 (48.80)	122.33 (44.53)	0.032
HbA1c (%) (mean [SD])	5.60 (1.00)	6.36 (1.36)	0.638	6.14 (1.41)	6.24 (1.33)	0.072
ALT (U/L) (mean [SD])	25.47 (17.54)	24.27 (12.25)	0.080	24.64 (16.38)	24.38 (12.28)	0.017
AST (U/L) (mean [SD])	25.03 (13.30)	24.86 (9.17)	0.014	25.40 (12.36)	24.98 (9.29)	0.039
TG (mg/dL), median (IQR)	103 (71–154)	121.5 (85–173)	0.122	117 (80–176)	122 (86–173)	0.025
TC (mg/dL) (mean [SD])	197.70 (41.23)	179.11 (41.22)	0.451	191.34 (38.55)	185.61 (41.27)	0.143
HDL‐C (mg/dL) (mean [SD])	53.25 (15.92)	52.52 (14.92)	0.047	53.76 (17.07)	53.20 (15.00)	0.035
LDL‐C (mg/dL) (mean [SD])	118.60 (35.87)	97.89 (33.70)	0.595	108.83 (32.30)	103.38 (33.93)	0.164
eGFR (mL/min/1.73 m^2^) (mean [SD])	98.76 (21.21)	79.02 (20.95)	0.936	80.67 (21.90)	81.04 (20.31)	0.017

Abbreviations: ALT, alanine aminotransferase; AST, aspartate aminotransferase; BMI, body mass index; eGFR, estimated glomerular filtration rate; FPG, fasting plasma glucose; HbA1c, hemoglobin A1c; HDL‐C, high‐density lipoprotein cholesterol; IQR, interquartile range; LDL‐C, low‐density lipoprotein cholesterol; SD, standard deviation; SMD, standardized mean difference; TC, total cholesterol; TG, triglycerides; WC, waist circumference.

### 3.3. Association of Statin Treatment With Mortality in Participants With CKMS

**Figure 2:**
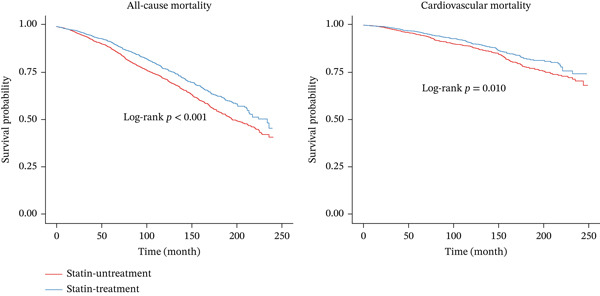
The Kaplan–Meier curve for all‐cause and cardiovascular mortalities according to statin treatment in the matched cohort.

In the matched cohort (*n* = 4762), statin‐treated participants exhibited significantly lower all‐cause mortality (21.0% vs. 29.5%, *p* < 0.001) and cardiovascular mortality (7.9% vs. 10.4%, *p* = 0.003) compared to statin‐untreated participants (Figure S5). Figure 2 displays the Kaplan–Meier survival curves for all‐cause and cardiovascular mortalities in the matched cohort. Univariable analysis showed HRs of 0.77 (95% CI 0.68–0.86, *p* < 0.001) for all‐cause mortality and 0.78 (95% CI 0.64–0.94, *p* = 0.010) for cardiovascular mortality among statin users. After multivariable adjustment, these protective associations remained statistically significant, with HRs of 0.73 (95% CI 0.65–0.82, *p* < 0.001) for all‐cause mortality and 0.74 (95% CI 0.61–0.90, *p* = 0.002) for cardiovascular mortality (Table 2).

**Table 2:** Cox regression model for the association between statin treatment and mortality in individuals with CKMS.

	Number of patients	Number of events	Unadjusted HR (95% CI)	Adjusted HR (95% CI)
All‐cause mortality				
Without statin	2381	703	Reference	Reference
With statin	2381	501	0.77 (0.68–0.86)	0.73 (0.65–0.82)
Cardiovascular mortality				
Without statin	2381	247	Reference	Reference
With statin	2381	187	0.78 (0.64–0.94)	0.74 (0.61–0.90)

*Note:* Cox proportional hazards model was adjusted for DBP, diabetes, diabetes medications, HbA1c, TC, and LDL‐C.

Abbreviations: DBP, diastolic blood pressure; HbA1c, hemoglobin A1c; TC, total Cholesterol; LDL‐C, low‐density lipoprotein cholesterol; HR, hazard ratio; CI, conﬁdence interval; CKMS, cardiometabolic‐kidney‐metabolic syndrome

Additionally, we further evaluated the prognostic value of statins in individuals with different stages of CKMS. As shown in Table S4 and Figure S6, among the matched cohort (*n* = 4762), 26.02% (*n* = 1239) were classified as having nonadvanced CKMS. Within this subgroup, 46.33% (*n* = 574) received statin therapy. The analysis revealed significantly lower all‐cause mortality in the statin‐treated group compared to the statin‐untreated group (7.1% vs. 10.7%, *p* = 0.039), while no statistically significant difference was observed in cardiovascular mortality (2.3% vs. 2.0%, *p* = 0.857). The majority of participants (73.98%, *n* = 3523) had advanced CKMS, among whom 51.29% (*n* = 1807) were treated with statins. Compared to untreated individuals, statin‐treated individuals exhibited significantly lower risks of all‐cause mortality (25.5% vs. 36.8%, *p* < 0.001) and cardiovascular mortality (9.6% vs. 13.6%, *p* < 0.001). Figure S7 displays the Kaplan–Meier survival curves for all‐cause and cardiovascular mortalities in individuals with different CKMS stages. The interaction between CKMS stage and statin use did not reach statistical significance for either all‐cause mortality (*p* for interaction = 0.81) or cardiovascular mortality (*p* for interaction = 0.13). However, statin therapy in individuals with nonadvanced CKMS stage showed no statistical trend toward reduced risk of all‐cause death (adjusted HR 0.71, 95% CI 0.48–1.05) and cardiovascular death (adjusted HR 1.14, 95% CI 0.53–2.47). In contrast, statin therapy in individuals with advanced CKMS stage was associated with a significant 30% reduction in all‐cause mortality (adjusted HR 0.70, 95% CI 0.62–0.79) and a 33% lower risk of cardiovascular mortality (adjusted HR 0.67, 95% CI 0.55–0.82) (Table S4).

### 3.4. Effects of Different Statin Treatments on Mortality

Among the 2831 participants receiving statin therapy, monotherapy regimens included atorvastatin (*n* = 843), simvastatin (*n* = 854), fluvastatin (*n* = 30), lovastatin (*n* = 179), pravastatin (*n* = 287), and rosuvastatin (*n* = 178), while pitavastatin was used in 6 patients. Four participants received dual statin therapy.

**Figure 3:**
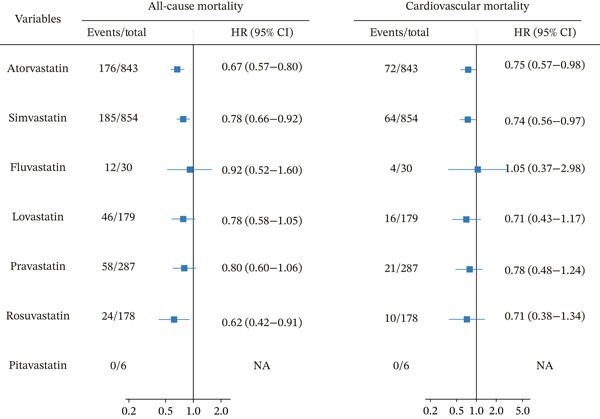
Forest plot of the association between different statin treatments and mortality in individuals with CKMS. Cox proportional hazards model was adjusted for DBP, diabetes, diabetes medications, HbA1c, TC, and LDL‐C. Abbreviations: DBP, diastolic blood pressure; HbA1c, hemoglobin A1c; TC, total cholesterol; LDL‐C, low‐density lipoprotein cholesterol; HR, hazard ratio; CI, confidence interval; CKMS, cardiovascular–kidney–metabolic syndrome.

Figure 3 compares mortality risks between statin‐treated and statin‐untreated CKMS participants, stratified by statin type. Atorvastatin (*n* = 843) demonstrated protection, associated with a 33% lower risk of all‐cause mortality (adjusted HR = 0.67, 95% CI 0.57–0.80) and a 25% lower risk of cardiovascular mortality (adjusted HR = 0.75, 95% CI 0.57–0.98). Rosuvastatin use (*n* = 178) was associated with the lowest risk of all‐cause mortality (adjusted HR = 0.62, 95% CI 0.42–0.91), whereas no significant association was observed for cardiovascular mortality (adjusted HR = 0.71, 95% CI 0.38–1.34). Simvastatin (*n* = 854) exhibited moderate benefits for both endpoints (all‐cause: adjusted HR = 0.78, 95% CI 0.66–0.92; cardiovascular: adjusted HR = 0.74, 95% CI 0.56–0.97), whereas fluvastatin (*n* = 30) and pitavastatin (*n* = 6) showed no significant effects due to limited sample sizes. Although lovastatin and pravastatin suggested mortality reduction trends, neither reached statistical significance. These findings suggested significant heterogeneity in the prognostic effects of different statins among individuals with CKMS. Specifically, atorvastatin, rosuvastatin, and simvastatin showed statistically significant reductions in all‐cause mortality, while atorvastatin and simvastatin additionally were associated with risk reduction of cardiovascular death. These results may provide important clinical reference for the selection of statin therapy in this patient population.

### 3.5. Subgroup Analysis

**Figure 4:**
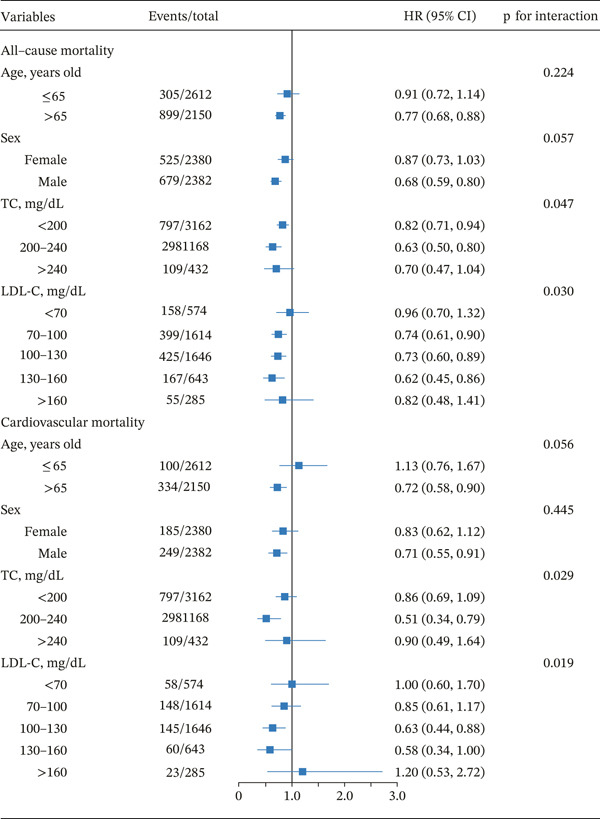
Forest plot of subgroup analysis of the association between statin treatment and mortality in individuals with CKMS. Cox proportional hazards model was adjusted for DBP, diabetes, diabetes medications, HbA1c, TC, and LDL‐C. The stratification variable was not included in the model. Abbreviations: DBP, diastolic blood pressure; HbA1c, hemoglobin A1c; TC, total cholesterol; LDL‐C, low‐density lipoprotein cholesterol; HR, hazard ratio; CI, confidence interval; CKMS, cardiovascular–kidney–metabolic syndrome.

Figure 4 presents subgroup analyses evaluating the association between statin therapy and mortality in individuals with CKMS. No significant age‐ or sex‐based interactions were detected (all *p* for interactions > 0.05). However, significant effect modifications by baseline lipid levels were observed, with notable interaction effects for both TC (*p* − interaction = 0.047 for all‐cause mortality and 0.029 for cardiovascular mortality) and LDL‐C (*p* − interaction = 0.03 and 0.019, respectively). For all‐cause mortality, maximal benefit emerged in patients with TC 200–240 mg/dL (HR = 0.63), whereas those with TC < 200 mg/dL (adjusted HR = 0.82) demonstrated attenuated effects, and those with TC > 240 mg/dL (adjusted HR = 0.70) showed no significant advantage. Similarly, LDL‐C 130–160 mg/dL conferred the greatest risk reduction (adjusted HR = 0.62), followed by 70–100 (adjusted HR = 0.74) and 100–130 mg/dL (adjusted HR = 0.73), while LDL − C < 70 (adjusted HR = 0.96) and > 160 mg/dL (adjusted HR = 0.82) exhibited null effects. Similar trends were noted for cardiovascular mortality. These results suggested that CKMS individuals with moderate TC and LDL‐C levels might indicate a better response to statin treatment in decreasing mortality.

### 3.6. Sensitivity Analysis

After excluding participants who died within 2 years of follow‐up, the all‐cause mortality was 20.50% (447/2180) in the statin‐treated group and 28.59% (623/2179) in the statin‐untreated group. The cardiovascular mortality was 7.75% (447/2180) in the statin‐treated group and 10.23% (623/2179) in the statin‐untreated group. Figure S8 shows the Kaplan–Meier curves for statin use and all‐cause and cardiovascular mortalities after excluding participants who died within 2 years of follow‐up. Univariable analysis showed HRs of 0.78 (95% CI 0.69–0.88, *p* < 0.001) for all‐cause mortality and 0.79 (95% CI 0.64–0.96, *p* = 0.019) for cardiovascular mortality among statin users. After adjusting for DBP, diabetes, diabetes medications, HbA1c, TC, and LDL‐C, these protective associations remained statistically significant, with HRs of 0.74 (95% CI 0.66–0.84, *p* < 0.001) for all‐cause mortality and 0.75 (95% CI 0.61–0.91, *p* = 0.004) for cardiovascular mortality (Table S5). Finally, *E* value analysis for all‐cause mortality (*E* value = 1.79) and cardiovascular mortality (*E* value = 1.77) based on the multivariable model showed that the observed associations could only be explained if unmeasured confounders had strong associations with both the exposure and the outcome.

## 4. Discussion

In the present study, the PSM analysis of NHANES 1999–2018 data demonstrated that statin use was associated with lower all‐cause mortality and cardiovascular mortality in individuals with CKMS, with numerically greater benefits observed in advanced CKMS stages, though statistical interaction was nonsignificant. There was heterogeneity in the effects of different statins, with atorvastatin, rosuvastatin, and simvastatin use associated with lower all‐cause mortality, while atorvastatin and simvastatin were linked to reduced cardiovascular mortality. TC and LDL‐C levels modified the associations between statin use and mortality, with a more pronounced benefit observed in patients with intermediate TC and LDL‐C levels compared to those with low or high levels. Collectively, our data support statin therapy as a promising strategy for CKMS management.

Current therapy of CKMS remains in transition from single‐disease approaches toward integrated care, primarily relying on combined therapies derived from existing guidelines for diabetes, CKD, and CVD (1). In metabolic regulation, sodium–glucose linked transporter 2 (SGLT2) inhibitors demonstrated well‐characterized cardiorenal protective effects. The EMPA‐REG OUTCOME trial showed that empagliflozin reduced cardiovascular mortality risk by 38% (13). The DAPA‐CKD trial further documented that dapagliflozin decreased the risk of renal composite endpoints by 39% in patients with CKD (14). A cohort study of 3657 Chinese patients with Stage 4 cardiovascular‐renal‐metabolic syndrome found that the use of SGLT2 inhibitors reduced the risk of major adverse cardiovascular events (MACEs) by 27.4%, the risk of cardiovascular death by 12.4%, the risk of major adverse kidney events (MAKE) by 11.5%, and the risk of all‐cause death by 11.3%, which supports its role in individuals with advanced CKMS stage (15). GLP‐1 receptor agonists also exhibit robust efficacy, with liraglutide in the LEADER trial reducing MACE risk by 13% (16) and semaglutide in the SUSTAIN‐6 trial demonstrating a 39% reduction in stroke risk (17). In the field of cardiovascular protection, statins remain the cornerstone therapy. A meta‐analysis by the Cholesterol Treatment Trialists’ (CTT) Collaboration demonstrated that each 1 mmol/L reduction in LDL‐C reduced vascular events by 22% (18). However, their efficacy and safety exhibit significant heterogeneity across age groups and genetic polymorphisms (19–23). In nephroprotection, renin–angiotensin–aldosterone system (RAAS) inhibitors exhibit well‐established efficacy. The IRMA‐2 trial demonstrated that irbesartan reduced the risk of diabetic nephropathy progression by 70% (24), while finerenone showed an 18% reduction in renal composite endpoints in FIDELIO‐DKD (25). Collectively, while current evidence has established the efficacy of organ/system‐specific therapeutic approaches, there remains insufficient evidence for integrated intervention strategies targeting the distinct metabolic‐cardio‐renal pathological network characteristic of CKMS.

The mortality of individuals with CKMS is obviously stage‐dependent, and the risk of death increases significantly with the increase of CKMS stage (26, 27). Effective management of CKMS prioritizes the prevention of cardiovascular complications (1, 28, 29). As a cornerstone of atherosclerotic cardiovascular disease (ASCVD) management, statin therapy has demonstrated clear benefits in reducing ASCVD risk in individual conditions, including CVD, diabetes, and CKD (30). The TNT study showed that intensive statin therapy significantly reduced MACE (including coronary heart disease death, nonfatal myocardial infarction, stroke, and resuscitation after cardiac arrest) by 22% in patients with stable coronary artery disease (31). The IDEAL study suggested that intensive statin therapy reduced the risk of nonfatal myocardial infarction by 17%, the risk of coronary revascularization by 31%, and the risk of peripheral arterial disease by 24% in patients with acute myocardial infarction (32). The SHARP trial demonstrated that simvastatin combined with ezetimibe reduced the risk of major vascular events by 17% in patients with renal insufficiency (33). The CARDS study confirmed that atorvastatin could reduce MACE risk by 37% and mortality by 27% in patients with Type 2 diabetes (34). A diabetic subgroup analysis of the MRC/BHF Heart Protection Study showed that simvastatin reduced the risk of a first MACE in diabetic patients by 22% (35). Notably, in addition to their traditional lipid‐lowering effects, statins could inhibit oxidative stress and improve endothelial progenitor cell dysfunction and apoptosis (36). Our study suggested that statin use was associated with significantly reduced all‐cause and cardiovascular mortalities in individuals with CKMS. Statistically significant associations were observed in individuals with advanced CKMS stages. However, the interaction between the CKMS stage and the statin effect was not statistically significant, indicating that these stage‐stratified findings should be interpreted as exploratory rather than as evidence of definitive staging specificity. In individuals with nonadvanced CKMS, point estimates consistently favored statin use but did not reach statistical significance, which may reflect lower event rates, limited statistical power, or shorter effective follow‐up rather than the absence of a treatment effect. Accordingly, further studies with longer follow‐up and adequate power are warranted to determine whether disease stage truly modifies treatment response. In addition to disease stage, we observed heterogeneity in the associations between different statin therapies and mortality outcomes. Atorvastatin, rosuvastatin, and simvastatin were significantly associated with reduced all‐cause mortality, whereas only atorvastatin and simvastatin showed statistically significant associations with cardiovascular mortality. However, these statin‐specific findings should be interpreted with caution. The sample sizes within individual statin subgroups were relatively small, and detailed information on statin dosage, treatment duration, and adherence was not available. Therefore, the observed differences may reflect prescribing patterns, baseline risk profiles, or other contextual clinical factors rather than true pharmacological heterogeneity. Accordingly, these analyses should be considered exploratory and hypothesis‐generating. Future head‐to‐head comparative studies with adequate power and detailed treatment data are required to determine whether statin‐specific differences in clinical outcomes represent genuine drug effects or contextual prescribing effects.

Epidemiological studies have demonstrated a rising prevalence of CKMS with notable sex differences in disease progression and prognosis. Although men exhibit a more pronounced increase in advanced CKMS stages, women consistently experience higher all‐cause mortality across stages, potentially reflecting greater vulnerability to metabolic stress and age‐related vascular dysfunction (37–40). Furthermore, age has been identified as an independent risk factor for the development and prognosis of CVD (41), diabetes and its complications (42), and CKD (43). Although there are significant age and gender differences in the severity and prognosis of CKMS, this study found that the mortality‐reducing effect of statin therapy in individuals with CKMS was not modified by these factors. This result is consistent with existing evidence‐based medicine for statins (44, 45). Furthermore, this study found that the mortality benefit of statin therapy varied according to baseline lipid levels. The greatest clinical benefit was observed among individuals with intermediate LDL‐C levels (130–160 mg/dL), corresponding to a 38% reduction in all‐cause mortality and a 42% reduction in cardiovascular mortality. TC levels showed a similar pattern, with the most pronounced benefit observed in the 200–240 mg/dL range. In contrast, among individuals with markedly elevated TC (> 240 mg/dL) or LDL‐C (> 160 mg/dL), substantial residual risk persisted despite statin therapy, suggesting that more intensive combination lipid‐lowering strategies may be required (46, 47). Conversely, attenuated benefit at very low lipid levels may reflect frailty or reverse causation. Although a modest residual imbalance in TC and LDL‐C remained after PSM, these biomarkers were explicitly adjusted for in all fully adjusted models to mitigate confounding by indication. The persistence of mortality benefit after lipid adjustment supports an association independent of baseline cholesterol levels, indicating that the observed lipid‐stratified differences more likely reflect effect modification rather than residual imbalance. Baseline lipid measurements, obtained prior to follow‐up, therefore provide important clinical context for interpreting treatment heterogeneity. Nevertheless, given the observational design, residual confounding cannot be entirely excluded, and these findings should be interpreted as hypothesis‐generating rather than causal.

Statins significantly reduce LDL‐C levels by inhibiting HMG‐CoA reductase, thereby effectively controlling the progression of atherosclerosis (48). Their anti‐inflammatory effects are primarily manifested by inhibiting the NF‐*κ*B and NLRP3 inflammasome pathways, helping to alleviate systemic inflammation (49). Furthermore, statins can upregulate eNOS expression and increase NO bioavailability, which not only improves endothelial function but also stabilizes plaques (50). Notably, these drugs also improve insulin sensitivity by regulating free fatty acid levels and activating the AMPK pathway (51, 52). These synergistic mechanisms suggest that statin therapy may intervene in abnormal cardiovascular, metabolic, and renal pathways in individuals with CKMS through multitarget regulation, thereby significantly improving clinical outcomes.

To our knowledge, this is the first study to investigate the effectiveness of statin therapy in reducing all‐cause and cardiovascular mortalities in individuals with CKMS. While providing crucial insights for statin application in CKMS management, several limitations warrant consideration. First, the retrospective design may introduce selection and information biases. Although we employed PSM and sensitivity analyses to mitigate these biases, residual confounding factors might persist. Future randomized controlled trials (RCTs) are necessary to establish causal relationships. Second, sample size limitations precluded robust head‐to‐head comparisons between different statin types, dosages, or treatment courses, which might differentially impact various CKMS stages. Self‐reported statin use may be underestimated. Finally, the predominantly US‐based NHANES cohort may limit the generalizability of these findings to other populations.

## 5. Conclusion

In this nationally representative cohort, statin use was associated with lower risks of all‐cause and cardiovascular mortalities among individuals with CKMS. Although no statistically significant interaction was observed between CKMS stage and statin use, the inverse associations were more evident among individuals with advanced CKMS stages, likely reflecting higher baseline risk and event rates. In addition, exploratory subgroup analyses suggested that the associations between statin use and mortality varied across baseline lipid levels, with more pronounced effects observed at intermediate TC and LDL‐C ranges. These findings support the potential clinical relevance of statin therapy in CKMS populations and highlight the need for adequately powered prospective studies to clarify treatment heterogeneity across disease stages, statin type, and different lipid profiles.

## Author Contributions

Cheng Yuan contributed to the conceptualization and design of the study, data collection and analysis, and drafting of the manuscript. Yuguo Ge, Wenqi Ge, Huaxue Wang, Ximing Deng, Jinmeng Chen, Kaixuan Niu, Jinhui Xiong, and Yang Xu contributed to the literature review, funding, and data interpretation. Kun Lu contributed to data analysis and interpretation of results and critical revision of the manuscript. Cheng Yuan and Yuguo Ge contributed equally to this work.

## Funding

This work was supported by Anhui Provincial University Scientific Research Project (Project Number 2022AH051527) for Ximing Deng and Natural Science Research Project of Anhui Educational Committee (Project Number: 2024AH051281) for Kun Lu.

## Disclosure

All authors have read and approved the final version of the manuscript. All authors agreed on the publication.

## Ethics Statement

The data used in this study were obtained from the NHANES database, which was established with the approval of the National Center for Health Statistics Institutional Review Board. Therefore, no additional ethical approval or written informed consent was required for this study. Please visit https://www.cdc.gov/nchs/nhanes/index.htm for more information.

## Conflicts of Interest

The authors declare no conflicts of interest.

## Supporting information


**Supporting Information** Additional supporting information can be found online in the Supporting Information section. Table S1 Definition of CKMS. Table S2 Methods for evaluating each CKMS stage. Table S3 Baseline characteristics before and after propensity score matching. Table S4 Cox regression model for the association between statin treatment and mortality among individuals with different CKMS stages. Table S5 Cox regression model for the association between statin treatment and mortality after excluding 403 individuals who die within 2 years of follow‐up. Figure S1 Percentage of missing data of each variable. Figure S2 Variance inflation factor of each variable in the adjusted model after the matched cohort. A variance inflation factor of < 5 for each variable suggested the absence of multicollinearity. Figure S3 Standardized mean differences in before and after propensity score matching. The matching improved variable balance, with an absolute SMD < 0.10. Figure S4 Distributional balance before and after propensity score matching. Figure S5 Impact of statin therapy on all‐cause and cardiovascular mortalities. Figure S6 Impact of statin therapy on all‐cause and cardiovascular mortalities, grouped by CKMS stage. Figure S7 The Kaplan–Meier curve for all‐cause and cardiovascular mortalities according to statin treatment among individuals with different CKMS stages. Figure S8 The Kaplan–Meier curves for all‐cause and cardiovascular mortalities according to statin treatment after excluding participants who died within 2 years.

## Data Availability

The data of the present study come from https://www.cdc.gov/nchs/nhanes/, and they are publicly available for free on the Internet. The data supporting the findings of the present paper could be provided by contacting the corresponding author without hesitation.
