# Supplementary material for: Effect of Statin Use on Mortality in Individuals With Cardiovascular–Kidney–Metabolic Syndrome: A Retrospective Propensity Score‐Matched Cohort Study
Source: Cardiovasc Ther. 2026 Mar 31;2026:7480081. doi: 10.1155/cdr/7480081 (PMC13140241; doi:10.1155/cdr/7480081)
Supplement: Supplementary file 1 — Supporting Information Additional supporting information can be found online in the Supporting Information section. Table S1 Definition of CKMS. Table S2 Methods for evaluating each CKMS stage. Table S3 Baseline characteristics before and after propensity score matching. Table S4 Cox regression model for the association between statin treatment and mortality among individuals with different CKMS stages. Table S5 Cox regression model for the association between statin treatment and mortality after excluding 403 individuals who die within 2 years of follow‐up. Figure S1 Percentage of missing data of each variable. Figure S2 Variance inflation factor of each variable in the adjusted model after the matched cohort. A variance inflation factor of < 5 for each variable suggested the absence of multicollinearity. Figure S3 Standardized mean differences in before and after propensity score matching. The matching improved variable balance, with an absolute SMD < 0.10. Figure S4 Distributional balance before and after propensity score matching. Figure S5 Impact of statin therapy on all‐cause and cardiovascular mortalities. Figure S6 Impact of statin therapy on all‐cause and cardiovascular mortalities, grouped by CKMS stage. Figure S7 The Kaplan–Meier curve for all‐cause and cardiovascular mortalities according to statin treatment among individuals with different CKMS stages. Figure S8 The Kaplan–Meier curves for all‐cause and cardiovascular mortalities according to statin treatment after excluding participants who died within 2 years. [file CDR-2026-7480081-s001.docx]

**Supplementary Materials**

Supplementary Table 1 Definition of CKMS.

Supplementary Table 2 Methods for evaluating each CKMS stage.

Supplementary Table 3 Baseline characteristics before and after propensity score matching.

Supplementary Table 4 Cox regression model for the association between statin treatment and mortality among individuals with different CKMS stages.

Supplementary Table 5 Cox regression model for the association between statin treatment and mortality in after excluding 403 individuals who die within 2 years follow-up.

Supplementary Figure 1 Percentage of missing data of each variable.

Supplementary Figure 2 Variance inflation factor of each variable in the adjusted model after the matched cohort. A variance inflation factor of < 5 for each variable suggested the absence of multicollinearity.

Supplementary Figure 3 Standardized mean differences in before and after propensity score matching. The matching improved variable balance, with an absolute SMD < 0.10.

Supplementary Figure 4 Distributional balance before and after propensity score matching.

Supplementary Figure 5 Impact of statin therapy on all-cause and cardiovascular mortality.

Supplementary Figure 6 Impact of statin therapy on all-cause and cardiovascular mortality, grouped by CKMS stage.

Supplementary Figure 7 Kaplan-Meier curve for all-cause and cardiovascular mortality according to statin treatment among individuals with different CKMS stages.

Supplementary Figure 8 Kaplan-Meier curves for all-cause and cardiovascular mortality according to statin-treatment after excluding participants who died within 2 years.

Supplementary Table 1 Definition of CKMS.

| CKMS conditions | Definition | CKMS indicators | Threshold for CKMS indicators |
| --- | --- | --- | --- |
| CVD | Individuals with clinical CVD or subclinical CVD | Clinical CVD | History of chronic heart failure, coronary heart disease, heart attack, or stroke |
|  |  | Subclinical CVD | Any of the following criterion is met:  1) Very high-risk CKD in KDIGO classification: UACR ≥ 300 mg/g and eGFR ≤ 45-59 ml/min/1.73m^2^, UACR ≥ 30 mg/g and eGFR ≤ 30-44 ml/min/1.73m^2^ , or eGFR ≤ 29 ml/min/1.73m^2^.  2) Predicted 10-year CVD risk ≥ 20% (PREVENT equations)* |
| Kidney  diseases | Individuals with CKD | CKD | Moderate-to-high-risk CKD in KDIGO classification: UACR ≥ 30 mg/g and eGFR ≥ 60 ml/min/1.73m^2^, UACR < 300 mg/g and eGFR ≤ 45-59 ml/min/1.73m^2^ , or UACR < 30 mg/g and eGFR ≤ 30-44 ml/min/1.73m^2^. |
| Metabolic disorders | Individuals with overweight/obesity, abdominal obesity, prediabetes,  diabetes, hypertension,  hypertriglyceridemia or MetS | Overweight/obesity | BMI ≥25 kg/m^2^ (or ≥23 kg/m2 if Asian ancestry)* |
|  |  | Abdominal obesity | Waist circumference ≥88/102 cm in female/male (or if Asian ancestry ≥80/90 cm in female/male) |
|  |  | Prediabetes | Fasting blood glucose ≥ 100-124 mg/dL or HbA1c ≥ 5.7%-6.4% and without self-reported diagnosis of diabetes, use of insulin, or oral hypoglycemic agents |
|  |  | Diabetes | Fasting blood glucose ≥ 125 mg/dL or HbA1c ≥ 6.5% or self-reported diagnosis of diabetes, use of insulin, or oral hypoglycemic agents |
|  |  | Hypertension | SBP ≥130 mm Hg or DBP ≥80 mmHg or self-reported diagnosis of hypertension or use of antihypertensive medications |
|  |  | Hypertriglyceridemia | Triglycerides ≥ 135 mg/dL |
|  |  | MetS | MetS is defined by the presence of 3 or more of the following:  1) Waist circumference ≥88/102 cm in female/male (or if Asian ancestry ≥80/90 cm in female/male).  2) HDL cholesterol ≥50/40 mg/dL in female/male.  3) Triglycerides ≥150 mg/dL.  4) Elevated blood pressure (SBP ≥130 mmHg or DBP ≥80 mmHg and/or use of antihypertensive medications)  5) Fasting blood glucose ≥100 mg/dL |

BMI, body mass index; CKD, chronic kidney disease; CKMS, cardiovascular-kidney-metabolic syndrome; CVD, cardiovascular disease; DBP, diastolic blood pressure; eGFR, estimated glomerular filtration rate; HDL, high-density lipoprotein; KDIGO, The Kidney Disease: Improving Global Outcomes; MetS, metabolic syndrome; SBP, systolic blood pressure; UACR, urinary albumin to creatinine ratio.

*10-year cardiovascular risk was estimated with the AHA Predicting Risk of CVD EVENTs (PREVENT) equations.High risk was defined as ≥20% 10-year CVD risk (based on recommended thresholds [https://professional.heart.org/en/guidelines and-statements/prevent-calculator]).

*Asian was not listed as a separate race/ethnicity until NAHNES 2011-2012, therefore the uniform threshold for BMI and waist circumference was used in all participants in NHANES 1999-2010.

Supplementary Table 2 Methods for evaluating each CKMS stage.

| CKMS stages | Definition | Criterion | Threshold for CKMS conditions |
| --- | --- | --- | --- |
| Stage 0: No  CKM risk factors | Individuals with normal BMI and waist circumference, normoglycemia,  normotension, a normal lipid profile, and no evidence of CKD or subclinical or clinical CVD | All criteria are met | BMI ≥25 kg/m^2^ (or ≥23 kg/m^2^ if Asian ancestry)* |
|  |  |  | Waist circumference <88/102 cm in female/male (or if Asian ancestry <80/90 cm in female/male) |
|  |  |  | Participants who do not meet the criteria for other stages |
| Stage 1: Excess or dysfunctional adiposity | Individuals with overweight/obesity,  abdominal obesity, or dysfunctional  adipose tissue, without the presence of other metabolic risk factors or CKD | Any of the three criteria is met | Overweight/obesity |
|  |  |  | Abdominal obesity |
|  |  |  | Prediabetes |
|  |  | All criteria are met | SBP <130 mm Hg and DBP <80 mm Hg without self-reported diagnosis of hypertension or use of antihypertensive medications |
|  |  |  | HDL cholesterol <50/40 mg/dL in female/male and triglycerides <150 mg/dL |
|  |  |  | Low-risk CKD in KDIGO classification according to eGFR and UACR: UACR < 30 mg/g and eGFR ≥ 60 ml/min/1.73m2. |
|  |  |  | Predicted 10-year CVD risk < 20% |
|  |  |  | No clinical CVD |
| Stage 2:  Metabolic risk  factors and CKD | Individuals with metabolic risk factors (hypertriglyceridemia, hypertension, MetS, diabetes), or CKD | Any of the five criteria is met | Hypertriglyceridemia |
|  |  |  | Hypertension |
|  |  |  | diabetes |
|  |  |  | MetS |
|  |  |  | Moderate-to-high-risk CKD in KDIGO classification |
|  |  | All criteria are met | No very high-risk CKD in KDIGO classification |
|  |  |  | Predicted 10-year CVD risk < 20% |
|  |  |  | No clinical CVD |
| Stage 3:  Subclinical CVD in CKMS | Subclinical CVD among individuals with excess/dysfunctional adiposity, other metabolic risk factors, or CKD | Any of the two criteria is met | Very high-risk CKD in KDIGO classification |
|  |  |  | Predicted 10-year CVD risk ≥ 20% |
|  |  | Any of the eight criteria is met | Overweight/obesity |
|  |  |  | Abdominal obesity |
|  |  |  | Prediabetes |
|  |  |  | Hypertriglyceridemia |
|  |  |  | Hypertension |
|  |  |  | diabetes |
|  |  |  | MetS |
|  |  |  | Moderate-to-high-risk CKD in KDIGO classification |
|  |  | The criterion is met | No clinical CVD |
| Stage 4: Clinical CVD in CKMS | Clinical CVD among individuals with excess/dysfunctional adiposity,  other metabolic risk factors, or CKD | The criterion is met | Clinical CVD |
|  |  | Any of the nine criteria is met | Overweight/obesity |
|  |  |  | Abdominal obesity |
|  |  |  | Prediabetes |
|  |  |  | Hypertriglyceridemia |
|  |  |  | Hypertension |
|  |  |  | diabetes |
|  |  |  | MetS |
|  |  |  | Moderate-to-high-risk CKD in KDIGO classification |
|  |  |  | Very high-risk CKD in KDIGO classification |

BMI, body mass index; CKD, chronic kidney disease; CKMS, cardiovascular-kidney-metabolic syndrome; CVD, cardiovascular disease; DBP, diastolic blood pressure; eGFR, estimated glomerular filtration rate; HDL, high-density lipoprotein; KDIGO, The Kidney Disease: Improving Global Outcomes; SBP, systolic blood pressure; UACR, urinary albumin to creatinine ratio.

*Asian was not listed as a separate race/ethnicity until NAHNES 2011-2012, therefore the uniform threshold for BMI and waist circumference was used in all participants in NHANES 1999-2010.


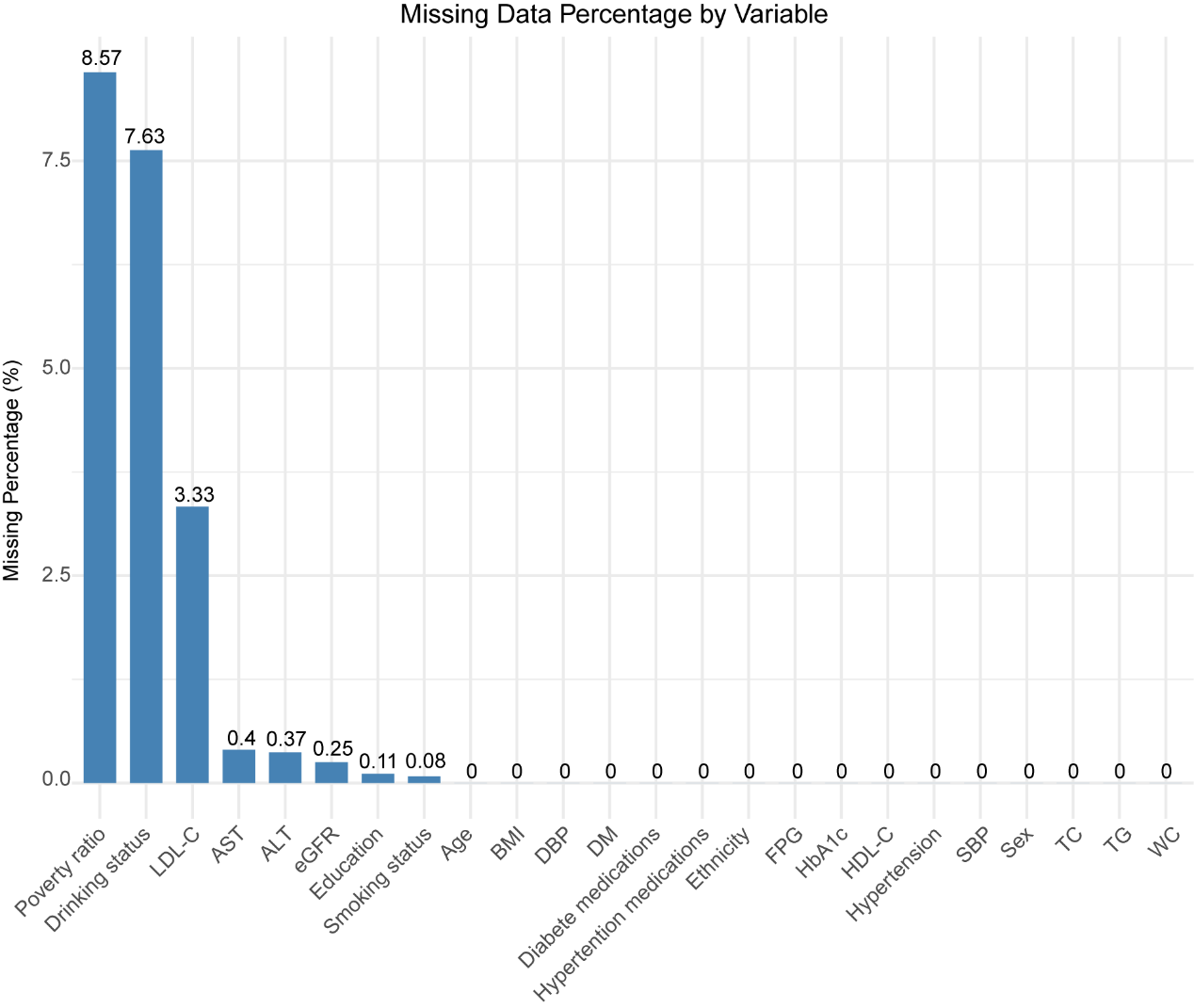


Supplementary Figure 1 Percentage of missing data of each variable.


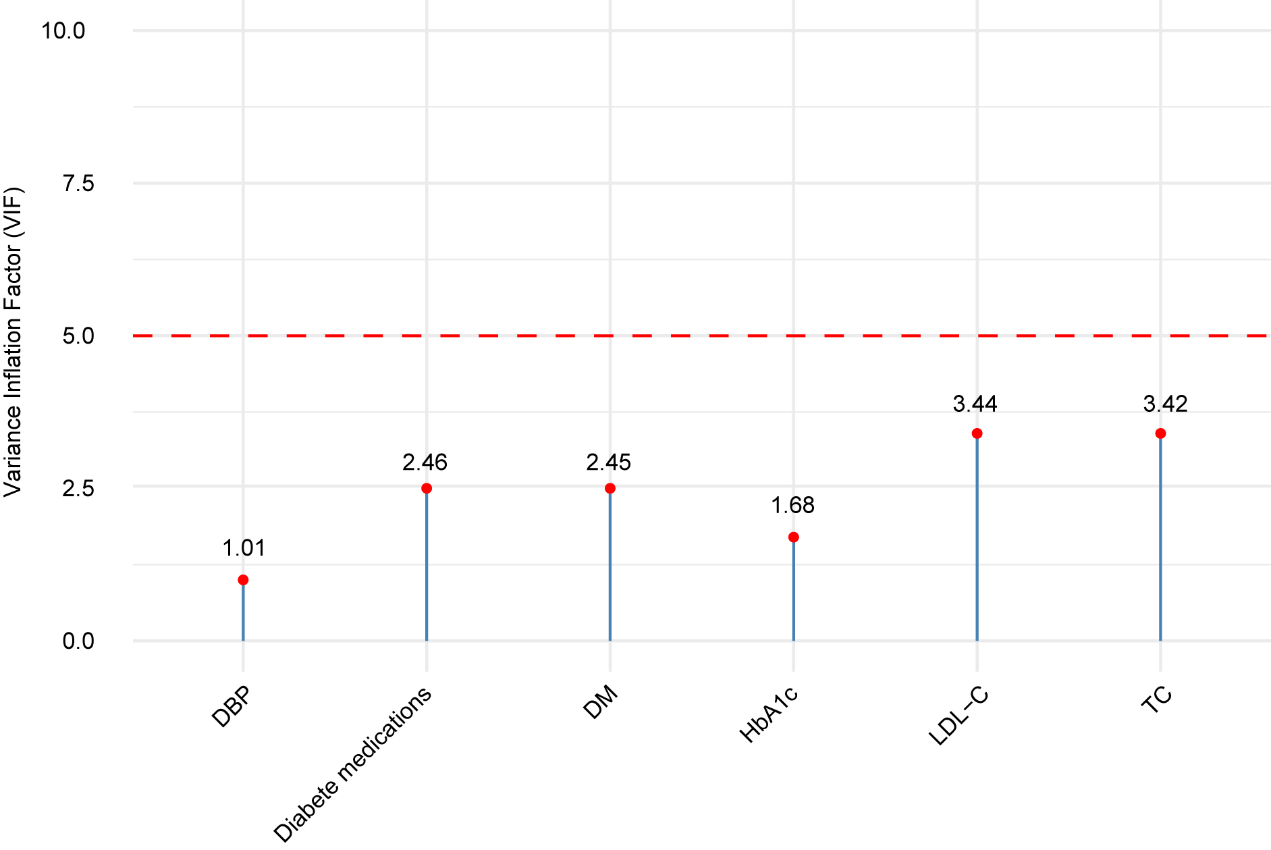


Supplementary Figure 2 Variance inflation factor of each variable in the adjusted model after the matched cohort. A variance inflation factor of < 5 for each variable suggested the absence of multicollinearity.

Supplementary Table 3 Baseline characteristics before and after propensity score matching.

| Variables | Before propensity score matching | | | After propensity score matching | | |
| --- | --- | --- | --- | --- | --- | --- |
|  | Statin-untreated group | Statin-treated group | *P* | Statin-untreated group | Statin-treated group | *P* |
|  | N=16139 | N=2948 |  | N=2381 | N=2381 |  |
| Age, years old, (mean (SD)) | 45.56 (16.64) | 64.41 (11.30) | <0.001 | 63.35 (13.54) | 62.96 (11.39) | 0.277 |
| Male, n (%) | 8055 (49.9) | 1553 (52.7) | 0.006 | 1165 (48.9) | 1217 (51.1) | 0.139 |
| Ethnicity, n (%) |  |  | <0.001 |  |  | 0.745 |
| White | 6637 (41.1) | 1467 (49.8) |  | 1168 (49.1) | 1178 (49.5) |  |
| Black | 3321 (20.6) | 565 (19.2) |  | 477 (20.0) | 478 (20.1) |  |
| Mexican | 3205 (19.9) | 374 (12.7) |  | 344 (14.4) | 319 (13.4) |  |
| Other | 2976 (18.4) | 542 (18.4) |  | 392 (16.5) | 406 (17.1) |  |
| Poverty-to-income ratio, (mean (SD)) | 2.50 (1.62) | 2.69 (1.61) | <0.001 | 2.67 (1.61) | 2.67 (1.63) | 0.968 |
| Education level, n (%) |  |  | <0.001 |  |  | 0.699 |
| Less than high school graduate | 4391 (27.2) | 838 (28.4) |  | 690 (29.0) | 669 (28.1) |  |
| High school graduate or general equivalency diploma | 3625 (22.5) | 740 (25.1) |  | 589 (24.7) | 582 (24.4) |  |
| Some college or above | 8123 (50.3) | 1370 (46.5) |  | 1102 (46.3) | 1130 (47.5) |  |
| Blood pressure, mmHg | |  |  |  |  |  |
| Systolic | 122.36 (18.36) | 129.96 (19.74) | <0.001 | 131.15 (20.63) | 130.02 (19.86) | 0.053 |
| Diastolic | 70.91 (11.70) | 68.08 (12.41) | <0.001 | 69.71 (13.07) | 68.85 (12.46) | 0.020 |
| Smoking status, n (%) |  |  | <0.001 |  |  | 0.528 |
| Never | 8977 (55.6) | 1398 (47.4) |  | 1181 (49.6) | 1142 (48.0) |  |
| Former | 3474 (21.5) | 1082 (36.7) |  | 819 (34.4) | 845 (35.5) |  |
| Now | 3688 (22.9) | 468 (15.9) |  | 381 (16.0) | 394 (16.5) |  |
| Drinking status, n (%) |  |  | <0.001 |  |  | 0.508 |
| Never | 2194 (13.6) | 439 (14.9) |  | 375 (15.7) | 357 (15.0) |  |
| Former | 2503 (15.5) | 719 (24.4) |  | 580 (24.4) | 559 (23.5) |  |
| Now | 11442 (70.9) | 1790 (60.7) |  | 1426 (59.9) | 1465 (61.5) |  |
| BMI, kg/m^2^, (mean (SD)) | 28.59 (6.60) | 30.08 (6.27) | <0.001 | 30.04 (6.96) | 29.95 (6.27) | 0.646 |
| WC, cm, (mean (SD)) | 97.29 (15.90) | 104.33 (14.69) | <0.001 | 103.55 (16.04) | 103.60 (14.54) | 0.916 |
| Diabetes, n (%) | 2032 (12.6) | 1396 (47.4) | <0.001 | 878 (36.9) | 950 (39.9) | 0.034 |
| Hypertension, n (%) | 5526 (34.2) | 2179 (73.9) | <0.001 | 1714 (72.0) | 1712 (71.9) | 0.974 |
| Diabetes medications, n (%) | 878 (5.4) | 1044 (35.4) | <0.001 | 568 (23.9) | 659 (27.7) | 0.003 |
| Hypertension medications, n (%) | 3125 (19.4) | 2286 (77.5) | <0.001 | 1684 (70.7) | 1726 (72.5) | 0.188 |
| FPG, mg/dl, (mean (SD)) | 104.96 (33.04) | 125.23 (45.60) | <0.001 | 120.86 (48.80) | 122.33 (44.53) | 0.277 |
| HbA1c, %, (mean (SD)) | 5.60 (1.00) | 6.36 (1.36) | <0.001 | 6.14 (1.41) | 6.24 (1.33) | 0.013 |
| ALT, U/L, (mean (SD)) | 25.47 (17.54) | 24.27 (12.25) | <0.001 | 24.64 (16.38) | 24.38 (12.28) | 0.549 |
| AST, U/L, (mean (SD)) | 25.03 (13.30) | 24.86 (9.17) | 0.518 | 25.40 (12.36) | 24.98 (9.29) | 0.18 |
| TG, mg/dl, median (IQR) | 130.00 (114.35) | 145.01 (131.51) | <0.001 | 143.90 (106.08) | 147.01 (140.06) | 0.388 |
| TC, mg/dl, (mean (SD)) | 197.70 (41.23) | 179.11 (41.22) | <0.001 | 191.34 (38.55) | 185.61 (41.27) | <0.001 |
| HDL-C, mg/dl, (mean (SD)) | 53.25 (15.92) | 52.52 (14.92) | 0.022 | 53.76 (17.07) | 53.20 (15.00) | 0.233 |
| LDL-C, mg/dl, (mean (SD)) | 118.60 (35.87) | 97.89 (33.70) | <0.001 | 108.83 (32.30) | 103.38 (33.93) | <0.001 |
| eGFR, ml/min/1.73m², (mean (SD)) | 98.76 (21.21) | 79.02 (20.95) | <0.001 | 80.67 (21.90) | 81.04 (20.31) | 0.546 |

Abbreviations: BMI, body mass index; WC, waist circumference; FPG, fasting plasma glucose; HbA1c, hemoglobin A1c; ALT, alanine aminotransferase; AST, aspartate aminotransferase; TG, triglycerides; TC, total Cholesterol; HDL-C, high-density lipoprotein cholesterol; LDL-C, low-density lipoprotein cholesterol; eGFR, estimated glomerular filtration rate; IQR, interquartile range; SD, standard deviation; SMD, standardized mean difference.


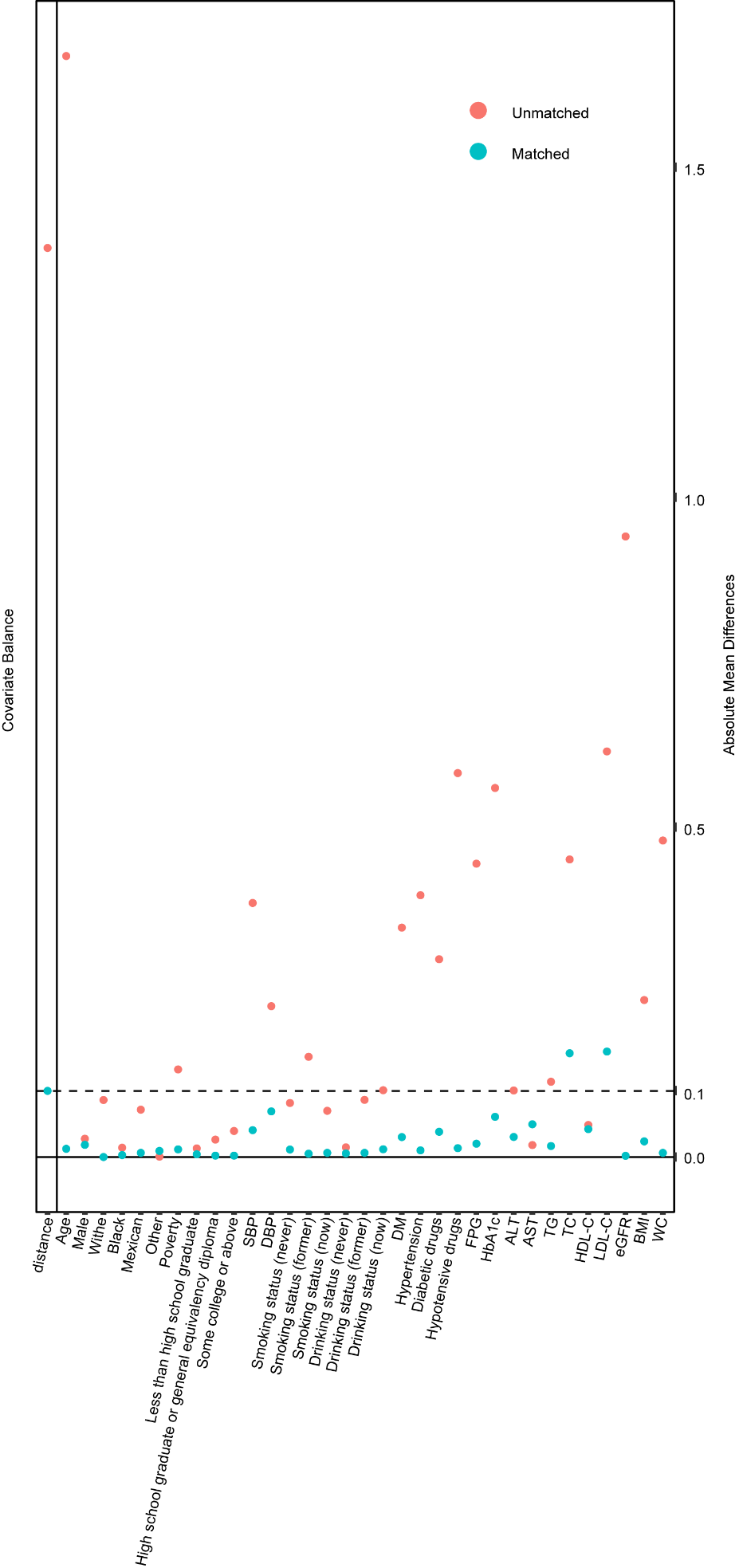


Supplementary Figure 3 Standardized mean differences in before and after propensity score matching. The matching improved variable balance, with an absolute SMD<0.10. Abbreviations: SBP, systolic blood pressure; DBP, diastolic blood pressure; BMI, body mass index; WC, waist circumference; FPG, fasting plasma glucose; HbA1c, hemoglobin A1c; ALT, alanine aminotransferase; AST, aspartate aminotransferase; TG, triglycerides; TC, total Cholesterol; HDL-C, high-density lipoprotein cholesterol; LDL-C, low-density lipoprotein cholesterol; eGFR, estimated glomerular filtration rate; DM, diabetes mellitus.


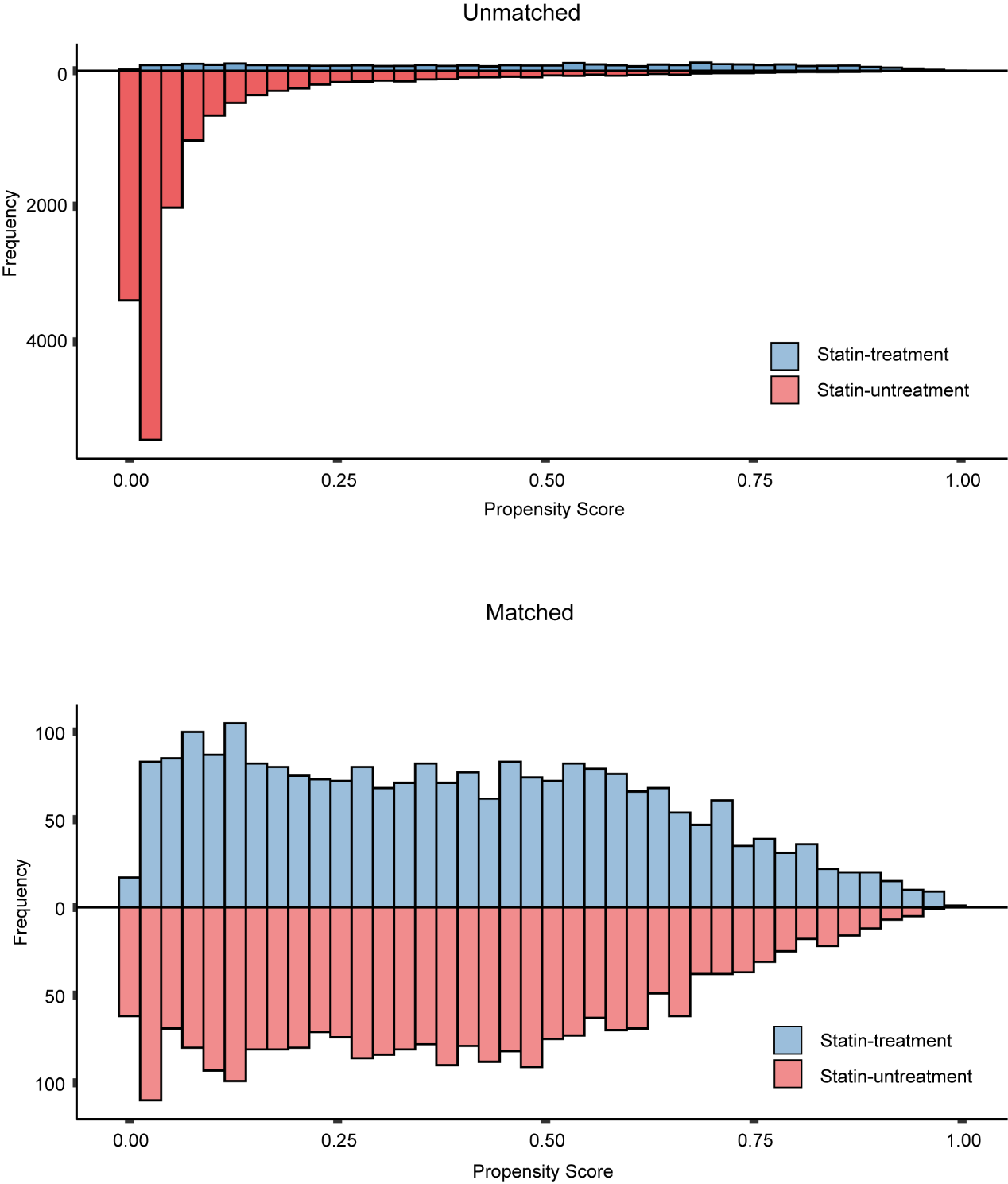


Supplementary Figure 4 Distributional balance before and after propensity score matching.


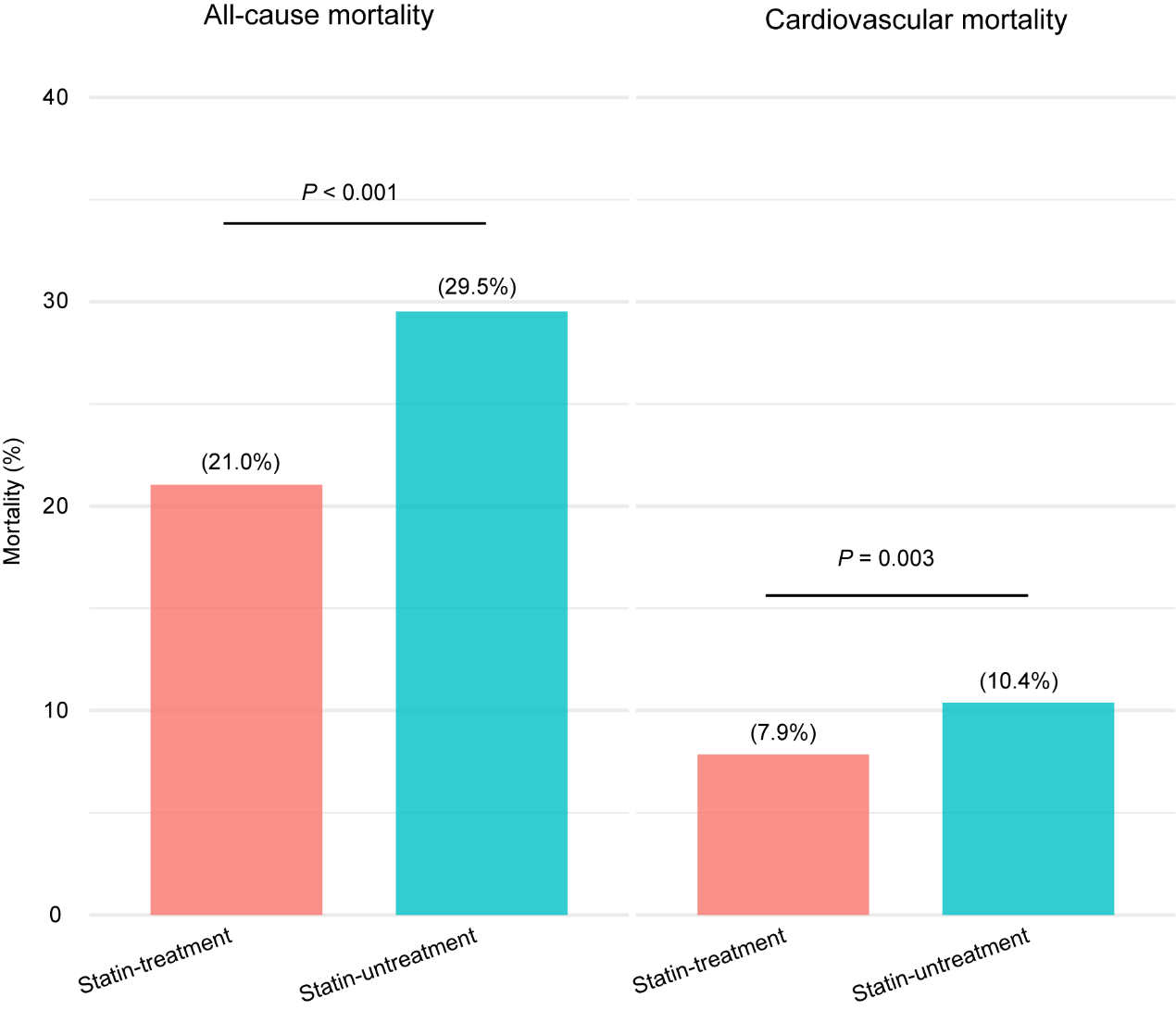


Supplementary Figure 5 Impact of statin therapy on all-cause and cardiovascular mortality.


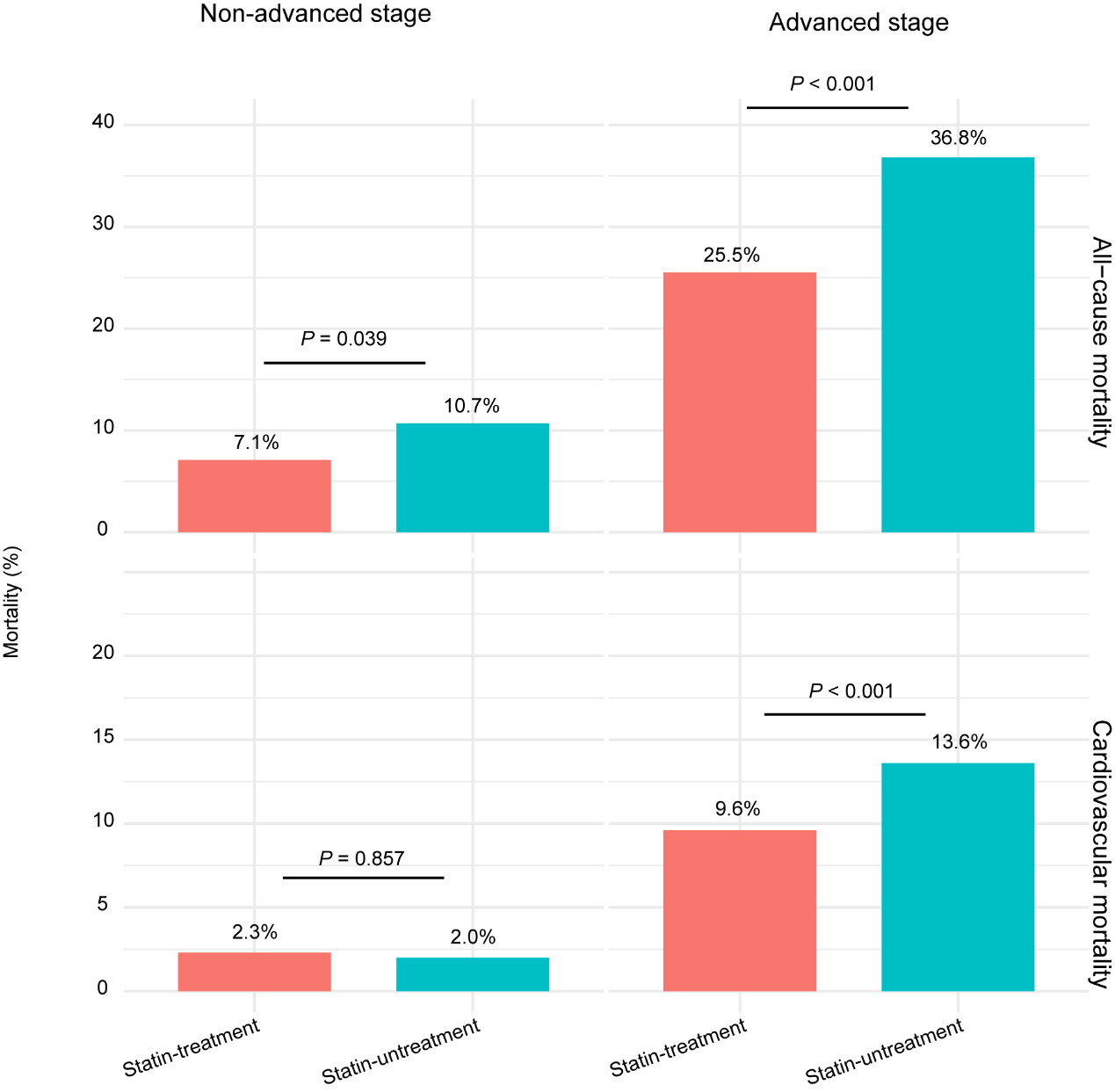


Supplementary Figure 6 Impact of statin therapy on all-cause and cardiovascular mortality, grouped by CKMS stage.


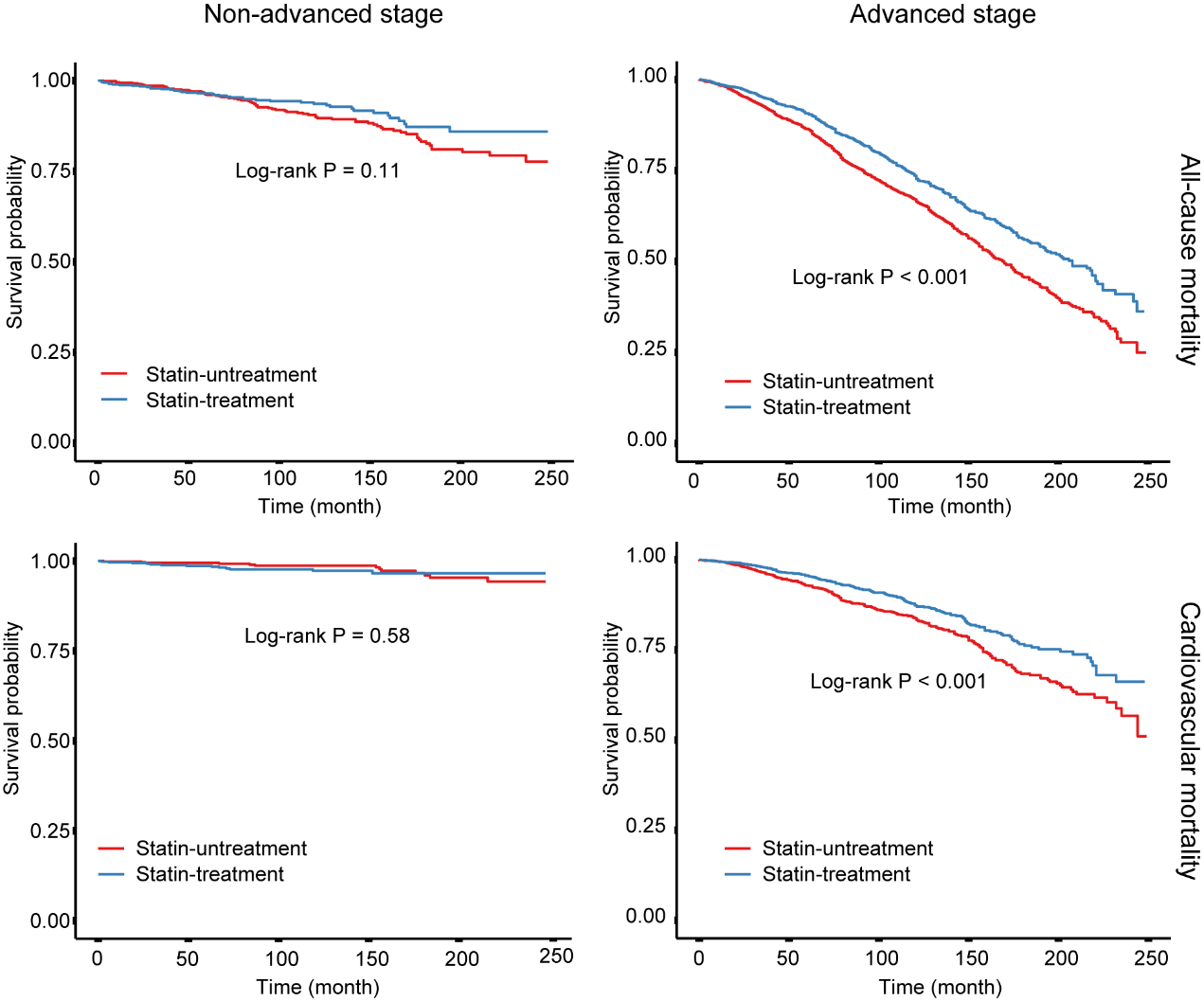


Supplementary Figure 7 Kaplan-Meier curve for all-cause and cardiovascular mortality according to statin treatment among individuals with different CKMS stages.

Supplementary Table 4 Cox regression model for the association between statin treatment and mortality among individuals with different CKMS stages.

|  | Number of patients | Number of events | Unadjusted HR (95% CI) | Adjusted HR (95% CI) |
| --- | --- | --- | --- | --- |
| No-advanced stage |  |  |  |  |
| All-cause mortality |  |  |  |  |
| Without statin | 665 | 71 | 1(Ref.) | 1(Ref.) |
| With statin | 574 | 41 | 0.73 (0.50-1.07) | 0.71 (0.48-1.05) |
| Cardiovascular mortality | |  |  |  |
| Without statin | 665 | 13 | 1(Ref.) | 1(Ref.) |
| With statin | 574 | 13 | 1.24 (0.59-2.61) | 1.14 (0.53-2.47) |
| Advanced stage |  |  |  |  |
| All-cause mortality |  |  |  |  |
| Without statin | 1,716 | 632 | 1(Ref.) | 1(Ref.) |
| With statin | 1,807 | 460 | 0.73 (0.64-0.82) | 0.70 (0.62-0.79) |
| Cardiovascular mortality | |  |  |  |
| Without statin | 1,716 | 234 | 1(Ref.) | 1(Ref.) |
| With statin | 1,807 | 174 | 0.70 (0.57-0.85) | 0.67 (0.55-0.82) |

Cox proportional hazards model was adjusted for DBP, diabetes, diabetes medications, HbA1c, TC, and LDL-C. Abbreviations: DBP, diastolic blood pressure; HbA1c, hemoglobin A1c; TC, total Cholesterol; LDL-C, low-density lipoprotein cholesterol; HR, hazard ratio; CI, conﬁdence interval; CKMS, cardiovascular-kidney-metabolic syndrome.


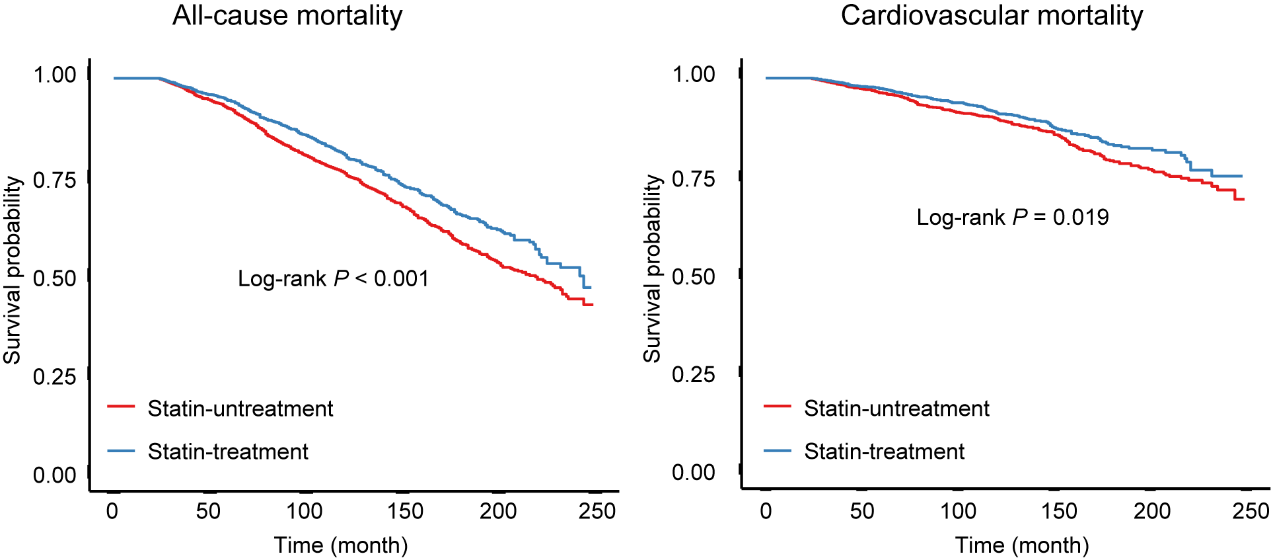


Supplementary Figure 8 Kaplan-Meier curves for all-cause and cardiovascular mortality according to statin-treatment after excluding participants who died within 2 years.

Supplementary Table 5 Cox regression model for the association between statin treatment and mortality in after excluding 403 individuals who die within 2 years follow-up.

|  | Number of patients | Number of events | Unadjusted HR (95% CI) | Adjusted HR (95% CI) |
| --- | --- | --- | --- | --- |
| All-cause mortality |  |  |  |  |
| Without statin | 2179 | 623 | 1(Ref.) | 1(Ref.) |
| With statin | 2180 | 447 | 0.78 (0.69-0.88) | 0.74 (0.66-0.84) |
| Cardiovascular mortality |  |  |  |  |
| Without statin | 2179 | 223 | 1(Ref.) | 1(Ref.) |
| With statin | 2180 | 169 | 0.79 (0.64-0.96) | 0.75 (0.61-0.91) |

Cox proportional hazards model was adjusted for DBP, diabetes, diabetes medications, HbA1c, TC, and LDL-C. Abbreviations: DBP, diastolic blood pressure; HbA1c, hemoglobin A1c; TC, total Cholesterol; LDL-C, low-density lipoprotein cholesterol; HR, hazard ratio; CI, conﬁdence interval.
